# Factors associated with prognostic or treatment outcomes in HIV/AIDS patients with and without hypertension in Eswatini

**DOI:** 10.1038/s41598-021-92185-0

**Published:** 2021-06-21

**Authors:** Sabelo Bonginkosi Dlamini, Hans-Uwe Dahms, Ming-Tsang Wu

**Affiliations:** 1 Department of Public Health, Kaohsiung Medical University, Kaohsiung, Taiwan; 2 Ph.D. Program in Environmental and Occupational Medicine and the Graduate Institute of Clinical Medicine, Kaohsiung Medical University, Kaohsiung, Taiwan; 3 Research Center for Environmental Medicine, Kaohsiung Medical University, Kaohsiung, Taiwan; 4 Department of Biomedical Science and Environmental Biology, Kaohsiung Medical University, Kaohsiung City, 807 Taiwan; 5 Department of Marine Biotechnology and Resources, National Sun Yat-Sen University, Kaohsiung City, 80704 Taiwan; 6 Department of Family Medicine, Kaohsiung Medical University Hospital, Kaohsiung Medical University, Kaohsiung, Taiwan; 7 Research Center for Environmental Medicine, Kaohsiung Medical University, Room 721, CS Building, 100 Shih-Chuan 1st Road, Kaohsiung, Taiwan

**Keywords:** Diseases, Risk factors

## Abstract

Non-communicable diseases are increasing faster in HIV/AIDS patients than in the general population. We studied the association between hypertension and other possible confounding factors on viral load and CD4-cell counts in hypertensive and non-hypertensive HIV/AIDS patients receiving antiretroviral therapy (ART) at a large hospital in Eswatini over a 4-year period. We performed a retrospective longitudinal review of the medical records of 560 ART patients divided into non-hypertension and hypertension groups (n = 325 and n = 235) from July 27 to September 8, 2018. Generalized Estimated Equation was used to analyze the longitudinal data. Hypertensive patients were more likely to have improved CD4-cell counts than non-hypertensive patients (OR = 1.83, [1.37–2.44]). ART patients with hypertension were more likely to have detectable viral loads, though not significant (OR = 1.37 [0.77–2.43]). In non-hypertensive patients, second line ART was significantly associated with viral load (OR = 8.61 [2.93–25.34]) and adverse side effects (OR = 3.50 [1.06–11.54]), while isoniazid preventive therapy was significantly associated with CD4-cell counts (OR = 1.68 [1.16–2.45]). In hypertensive patients, factors associated with viral load were WHO HIV stage (OR = 2.84 [1.03–7.85]) and adherence (OR = 8.08 [1.33–49.04]). In both groups, CD4-cell counts significantly and steadily increased over time (p-value < 0.001). Results show a significant association between hypertension and CD4 cell counts but not viral load. In ART patients with and without hypertension, the factors associated with prognostic markers were different. More attention may need to be paid to ART patients with well controlled HIV status to monitoring and controlling of hypertension status.

## Introduction

Non-communicable diseases (NCDs) are responsible for 41 million deaths worldwide each year, and about 75% of these deaths occur in the low- and middle-income countries (which include the Sub-Saharan Africa). Cardiovascular diseases account for a large proportion (17 million deaths annually)^1^. Hypertension, is a major risk factor for cardiovascular, cerebrovascular, and renal disorders, and directly contribute to about 58% deaths attributed to cardiovascular diseases^2^, hence for this study it has been chosen as a marker for NCDs. It is often observed among people living with HIV/AIDS (PLHIV), especially in low- and middle-income countries^3^ and has been associated with risk factors such as increased body weight, increasing age, ethnicity, hypersodic diet, alcohol abuse, sedentary lifestyle, unfavorable socioeconomic factors, genetic predisposition, and other cardiovascular risk factors^1,4–7^. Associations between markers of prognosis of HIV infection such as viral load, duration of infection and CD4-cell count have also been associated with cardiovascular diseases^8,9^.

Sub-Sahara Africa (and Africa in general) represents the most HIV/AIDS-affected part of the world, followed by Asia and the Pacific^10^. Almost 40 million (37.9) people were living with HIV in 2018, a year seeing 1.7 million new cases and 770,000 HIV/AIDS deaths worldwide^11^. Eswatini, located in the southern part of Africa, has the world’s highest prevalence of HIV/AIDS. In Eswatini 27% of the population being 15 years and older have HIV/AIDS^12^. However, the country has made great progress in the control of the disease. Based on the 2011 and 2017 household Swaziland HIV Incidence Measurement Surveys (SHIMS 1 and 2), which included blood sampling, Eswatini had reduced its incidence by half between 2011 and 2016 (2.4% vs. 1.70%)^12^.

HIV/AIDS incidence reduction can be attributed to the introduction of antiretroviral therapy (ART). Antiretroviral therapy has transformed HIV/AIDS from a catastrophic disease to a manageable chronic disease^13–15^. However, this increased survival puts PLHIV at greater risk to NCDs like among others hypertension and cardiovascular conditions^16^. While cardiovascular diseases have been associated with duration of infections as well as with viral load and CD4-cell counts (both prognostic markers for HIV)^9^, on the other hand, how the management of cardiovascular disease affects these prognostic markers for HIV are less known^17^. There has been several documentations or information in relation to the benefits of ART since its introduction into clinical practice. However, it remains unclear how well or how poorly NCDs are being managed in PLHIV^17–19^. It’s also unknown whether the co-existence of NCDs and HIV/AIDS will affect the progress of each other during their treatment. Nevertheless, there is evidence that the burden of NCDs like chronic cardiovascular conditions is increasing among PLHIV compared to the general population^16^. Due to the common co-existence of HIV/AIDS and NCDs, there is growing advocacy for integrating the healthcare of HIV/AIDS and NCDs. However, such a step is one of the most challenging as it requires the development, evaluation and promulgation of chronic care models that are culturally friendly, adoptable in resource-limited healthcare systems, and responsive to the local burden of diseases^20^.

In patients who have no resistant mutations and who adhere to treatment strategies, ART-viral load reduction is expected to be achieved within 8 to 24 weeks^21^. WHO recommends that the viral load should be tested around 6 months after the initiation of ART, and yearly thereafter if the viral load has been successfully reduced^22^. CD4-cell counts provide an overall picture of the immune status of PLHIV and are often used to determine health status and decide when to initiate and discontinue the use of medications to treat opportunistic infections^23^.

In 2014, the UNAIDS launched its Fast-Track Strategy, also known as 90 (tested)-90 (treated)-90 (successfully treated) treatment target, hoping to improve the response to the HIV epidemic by various low- and middle-income countries^24^. About 85% (84.7) of those responded to Swaziland HIV Incidence Measurement Survey 2 reported knowing their HIV status, 87.4% of those who were HIV positive reported receiving ART, and 91.9% of those on ART reported successful viral load suppression. Prevalence of viral load suppression in all adults aged 15 years and older who were HIV positive was 73.1%. Younger HIV-positive adults (15–24 years old) were found to have the lowest success rate of reduced viral loads (50.6%) compared to all adults who were ≥ 15 years old^12^.

No study has investigated the impact of NCDs on HIV-ART outcomes as yet, more especially in the Southern African region, especially with reference to hypertension. Therefore, we retrospectively reviewed 560 medical charts of one hospital treating PLHIV to identify those with and without hypertension and following the results of their annual viral load and CD4 cell count tests for 4 years and analyzed the data controlling for age, gender, treatment, adverse effects, adherence, and year.

## Material and methods

### Study design and sample

**Figure 1:**
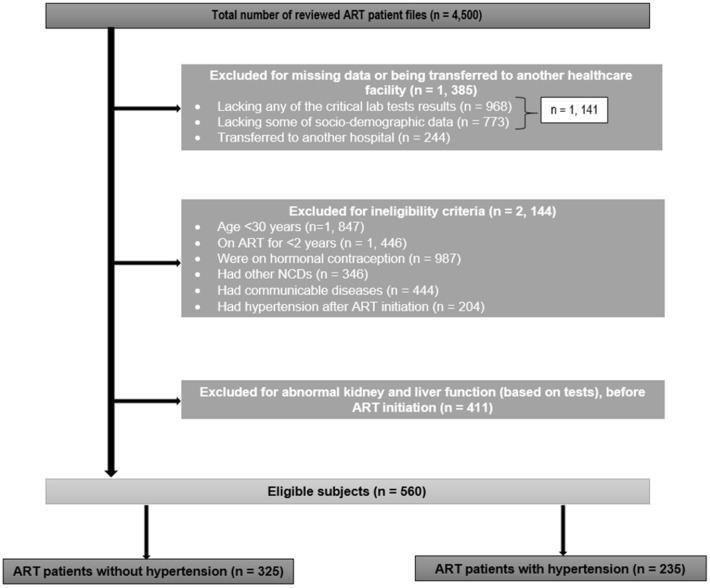
Flow chart for exclusion criteria and subject selection process.

In this longitudinal study, we reviewed the medical charts of PLHIV at Raleigh Fitkin Memorial (RFM) Hospital in the central part of Eswatini to follow changes in viral load and CD4 cell count in ART patients with and without hypertension over a 4-year study period. As seen in Fig. 1, we first identified more than 4500 patients receiving ART at the healthcare facility. To be included, patients had to provide the following characteristics: 30 years old or older, tested seropositive for HIV, being on ART for at least 2 years, have normal kidney and liver functions prior to ART, not taking any hormonal contraceptives, and be free of any other diseases other than HIV and physician diagnosed hypertension prior to ART. Those who had missing data and/or transferred to another hospital were excluded.

Patients 30 years old or older were the ones selected for this study because they are generally more susceptible to NCDs compared to the younger age groups. According to the WHO, 15 million of deaths attributed to NCDs occur between the ages of 30 and 69 years^1^. The reason for including patients who have been on ART for 2 years or more is that by such time, it is expected that ART would have a significant impact on the CD4-cell count and viral load. It is thus easy to determine the trend of the prognosis on the stipulated times of the study^25,26^. The main reason for excluding those who had hypertension after ART initiation is that we needed to exclude hypertension that might have resulted from ART, as it would have led to biasness in this study. The reason for excluding those who use hormonal therapy is that this factor is likely to be confounding for this study as it can directly affect the prognosis of HIV/AIDS, and/or hypertension according to some studies^27–30^.

After exclusion, we were left with 560 ART patients. They were divided into 325 non-hypertensive patients. In determining the normal kidney and liver function used as criteria for eligibility, different laboratory tests were used. For liver function, the AST and ALT are laboratory tests commonly used in Eswatini hospitals for PLHIV to determine their liver function, hence they were also used in this study. For kidney function, the serum creatinine (SCr) test was used for this study as it is the commonly used test to assess kidney function in PLHIV in Eswatini. The threshold for AST and ALT according to McAuley and Park et al*.,* is relatively 35 U/L^31,32^. For patients to be included in the study, their AST and ALT had to be < 35 U/L before ART initiation. The normal range for SCr is 45–90 µmol/L^33^. For patients to be included in the study, their SCr had to be < 90 µmol/L. This applied for both patients with hypertension and without hypertension.

### Dependent and independent variables

In this study, prognosis was not defined as survival but as improvement or worsening of the HIV disease status as defined by two dependent (outcome) variables: viral loads and CD4-cell counts. Better prognosis was defined by lower viral load, using the Southern African HIV Clinicians Society (SAHCS) 2017 Adult ART Guidelines’ threshold of defining undetectable viral loads (≤ 50 copies/mL), and a higher CD4-cell count which, according to WHO, would be a threshold of ≥ 500 cells/µL)^34,35^. The A worse prognosis was defined as persistently having a viral load above and CD4-cells count below these thresholds. SAHCS further states that viral load ≤ 50 copies/mL is associated with the most durable benefit. Hence such threshold has been chosen for this study. According to WHO, if a patient is progressing well in ART, their viral load has to be < 1000 copies/mL after a few months’ initiation to the treatment. The findings from PARTNER 1 and 2 study further stated that at < 200 c/mL, serodiscordent couples engaging in homosexual and heterosexual intercourse, chances of the virus transmission to the HIV negative partner was ZERO. In this study, the < 50 copies/mL from SAHCS is even a better threshold as it is less than < 200 copies/mL.

The main independent variable for this study was the presence of hypertension. Other independent variables were also included because they could possibly affect outcomes and confounding results. Based on our literature review, these were adherence, age, sex, isoniazid preventive therapy (IPT), residence or administrative region, WHO HIV staging, year of study, and ART adverse effects^14,35–39^. The adverse effects that were experienced by the patients included: drug-induced bone-marrow suppression, lactic acidosis, hepatic toxicity, pancreatitis, and peripheral neuropathy, fat maldistribution, body habitus changes, hyperlipidemia, hyperglycemia, insulin resistance, skin rashes and hypersensitivity reactions, nausea, diarrhea, dizziness, depression, myocardial infarction, ischemic stroke, proximal renal tubulopathy and elevated creatinine, and nephrolithiasis and cholelithiasis. Adherence was categorized in this study into underuse, close adherence and overuse of prescribed ART medications based on pill counts collected from the patients’ medical records. Pill count scores below 90% (underuse) were defined as poor adherence, those between 95 and 105% (close adherence) were defined as good adherence, and > 105% (overuse) were defined as excessive. Excessive adherence is associated with the patient taking the medication more than is expected, which can suggest an overdose or the fact that the patient might be sharing the medication with someone else. The patients were divided into three age ranges, 30–40 years, 41–50 years, and > 50 years.

### Data collection and management

The data from medical records were extracted using a 28-item standardized case-report form with three sections, one collecting socio-demographic data, another collecting medical history, and the other collecting laboratory results. The data were collected from July 27, 2018 to September 8, 2018 by the principal investigator. To ensure anonymity and confidentiality, no identifying information (such as names) was recorded. Instead, each patient was assigned an identification code to be matched anonymously with his or her data.

### Data analysis

Group continuous variables were analyzed descriptively and expressed as median with interquartile range (IQR) and mean ± standard deviation (SD) and categorical variables were expressed as frequency and percentages. Chi-square (χ^2^) test was used to compare proportional differences between different levels of categorical independent variables. For multivariate analysis, the Generalized Estimated Equations model was employed to study the relationship between independent variables and dependent variables in a whole group analysis and subgroup analysis. Odds ratios (OR) were reported with 95% confidence intervals (CI). *P*-values < 0.05 were considered significant. All statistical operations were performed using IBM SPSS version 22 (https://www.ibm.com/support/pages/downloading-ibm-spss-statistics-22).

### Ethical considerations

The protocol for this study was approved by the National Health Research Review Board (NHRRB) at the Eswatini Ministry of Health on July 24, 2018. The need for written consent was waived by the NHRRB because anonymity of the participants was ensured during the patients’ files review, and there was no direct contact or interaction with participants. All methods were carried out in accordance with the relevant guidelines and regulations which pertain to the use of humans as participants of a study.

## Results

### Participant characteristics

As can be seen in Table 1, the mean age of all the study participants was 44 years with SD of ± 8.61. For those with hypertension, their mean age was 47.3 years, with a SD of ± 9.44, yet those without hypertension, their mean age was 41.7 years, with SD of ± 7.12. Most study participants were 30–40 years old (44.8%). 33.4% were 41–50, and 29.4% were > 50. Fifty-six percent (56%) of the non-hypertensive patients were 30–40 years old. Of those who had hypertension, 36.2% were 41–50 years old and 34.5% were > 50. The difference in prevalence of hypertension among the three age groups was significant (*p-*value < 0.001).

**Table 1:** Socio-demographic characteristics of study subjects.

Parameters	Total (N = 560)	Presence of hypertension		*p*-value
		No (n = 325)	Yes (n = 235)	
**Age**				
30–40	251 (44.8%)	182 (56.0%)	69 (29.4%)	< 0.001*
41–50	187 (33.4%)	102 (31.4%)	85 (36.2%)	
> 50	122 (21.8%)	41 (12.6%)	81 (34.5%)	
Mean age ± SD	44.04 ± 8.61	41.71 ± 7.12	47.26 ± 9.44	
**Sex**				
Female	352 (62.9%)	197 (60.6%)	155 (66.0%)	0.192
Male	208 (37.1%)	128 (39.4%)	80 (34.0%)	
**Settlement**				
Rural	348 (62.1%)	210 (64.6%)	138 (58.7%)	0.086
Urban and peri-urban	212 (37.9%)	115 (35.4%)	97 (41.3%)	
**Region**				
Within Manzini	396 (70.7%)	220 (67.7%)	176 (74.9%)	0.038*
Outside Manzini	164 (29.3%)	105 (32.3%)	59 (25.1%)	

SD: standard deviation; N: total study population; n: subset of study population; > : greater than; < : less than.

*Statistically significant P-value.

As can be seen in Table 2, 94.8% of participants were diagnosed as having WHO HIV stage one disease with the remaining approximately 5% stages two, three, or four. Of those with hypertension, 91.5% had stage 1 disease. Those diagnosed having stages 2, 3, and 4 were amalgamated into a “stage 2 and higher” group. The stage one group had a significantly higher prevalence of hypertension than the amalgamated stage group (91.5% vs. 8.5%; Chi-square *P-*value = 0.003). Ninety-eight percent of all participants were receiving first line treatment. Those receiving first line treatment had a significantly higher prevalence of hypertension than those receiving second line treatment (99.6% vs. 0.4%; *p*-value = 0.03). Almost 3.8% of the participants (non-hypertensive 2.5%, hypertensive 5.5%) had adverse side effects to ART, with no significant difference between the two groups. Most of the hypertensive patients were taking diuretics (33.6%), and about 31.5% were on lifestyle modifications, which has to do with healthy habits like regular exercise, low fat and salt diet among others. About 19.1% of the participants were taking a combination of diuretics + ACE inhibitors or beta blockers.

**Table 2:** Clinical characteristics of study participants.

	Total (N = 560)	Presence of hypertension		*p*-value
		No (n = 325)	Yes (n = 235)	
**W.H.O. HIV staging**				
Stage 1	531 (94.8%)	316 (97.2%)	215 (91.5%)	0.003*
Stage 2–4	29 (5.2%)	9 (2.8%)	20 (8.5%)	
**INH preventive therapy**				
No	256 (45.7%)	154 (47.4%)	102 (43.4%)	0.352
Yes	304 (54.3%)	171 (52.6%)	133 (56.6%)	
**ART line of treatment**				
1st line	549 (98.0%)	315 (96.9%)	234 (99.6%)	0.028*
2nd line	11 (2.0%)	10 (3.1%)	4 (0.4%)	
**ART duration**				
2–4 years	256 (45.7%)	123 (37.8%)	102 (43.4%)	0.071
5–8 years	304 (54.3%)	202 (62.2%)	133 (56.6%)	
Mean ART duration ± SD	4.4 ± 1.8	5.2 ± 1.44	3.9 ± 2.1	
**Has adverse effects for ART**				
No	539 (96.3%)	317 (97.5%)	222 (94.5%)	0.064
Yes	21 (3.8%)	8 (2.5%)	13 (5.5%)	
**Had drug substitution**				
No	504 (90.0%)	299 (92.0%)	205 (87.2%)	0.039*
Yes	56 (10.0%)	26 (8.0%)	30 (12.8%)	
**Adherence**				
Poor	119 (21.3%)	72 (22.2%)	47 (20.0%)	0.758
Good	391 (69.8%)	223 (68.6%)	168 (71.5%)	
Excessive	50 (8.9%)	30 (9.2%)	20 (8.5%)	
**Hypertension treatment**				
None	325 (58.0%)	325 (100%)	0 (0.0%)	< 0.001
Lifestyle modification	74 (13.2%)	–	74 (31.5%)	
Diuretics	79 (14.1%)	–	79 (33.6%)	
ACE inhibitor	3 (0.5%)	–	3 (1.3%)	
Calcium channel blockers	1 (0.2%)	–	1 (0.4%)	
Diuretics + ACE inhibitor or beta blocker	45 (8.0%)	–	45 (19.1%)	
Diuretics + ACE inhibitor or beta blocker + calcium channel blocker	19 (3.4%)	–	19 (8.1%)	
Calcium channel blockers + ACE inhibitor or beta blocker	4 (0.7%)	–	4 (1.7%)	
Diuretics + calcium channel blocker	10 (1.8%)	–	10 (4.3%)	

N: total study population; n: subset of study population; INH: isoniazid; W.H.O.: World Health Organization; HIV: human immunodeficiency virus; ART: antiretroviral therapy; –: irrelevant.

*Statistically significant P-value.

### Viral loads and CD4-cell counts

Table 3 provides a summary of our two HIV related outcomes, which were successfully controlled viral load (< 50 copies/mL, controlled status) and healthy CD4-cell count (≥ 500 cells/µL), in non-hypertensive and hypertensive participants. A large majority in both groups had viral loads < 50 copies/mL over a 4-year period, ranging from 93.2 to 97.4% in non-hypertensive participants and 95.1% to 97.2% in those with hypertension. Median CD4-cell counts expressed along with interquartile (IQR) ranges were 398 (257–568) in 2015, 441 (302–613) in 2016, 476.5 (349–613) in 2017, and 521.5 (368–689) in 2018.

**Table 3:** HIV related outcomes.

	Without hypertension				With hypertension				TOTAL			
	Time (year)				Time (year)				Time (year)			
	2015 (n = 325)	2016 (n = 325)	2017 (n = 325)	2018 (n = 325)	2015 (n = 235)	2016 (n = 235)	2017 (n = 235)	2018 (n = 235)	2015 (N = 560)	2016 (N = 560)	2017 (N = 560)	2018 (N = 560)
**Viral load**												
< 50	315 (96.9%)	316 (97.2%)	309 (95.1%)	311 (95.7%)	229 (97.4%)	219 (93.2%)	225 (95.7%)	224 (95.3%)	544 (97.1%)	535 (95.5%)	534 (95.4%)	535 (95.5%)
≥ 50	10 (3.1%)	9 (2.8%)	16 (4.9%)	14 (4.3%)	6 (2.6%)	16 (6.8%)	10 (4.3%)	11 (4.7%)	16 (2.9%)	25 (4.5%)	26 (4.6%)	25 (4.5%)
Median (IQR)	10 (5–16)	11 (6–16)	11 (6–17)	11 (6–16)	10 (6–15)	11 (6–16)	12 (6–17)	12 (6–16)	10 (6–15)	11 (5–16)	11 (6–17)	11 (6–16)
**CD4-cell count**												
< 500	224 (73.9%)	217 (67.2%)	182 (56.3%)	162 (50.5%)	117 (53.4%)	117 (49.8%)	119 (50.6%)	92 (39.5%)	341 (65.3%)	334 (59.9%)	301 (53.9%)	254 (45.8%)
≥ 500	79 (26.1%)	106 (32.8%)	141 (43.7%)	159 (49.5%)	102 (46.6%)	118 (50.2%)	116 (49.4%)	141 (60.5%)	181 (34.7%)	224 (40.1%)	257 (46.1%)	300 (54.2%)
Median (IQR)	338 (234–514)	402 (284–569)	456 (330–617)	496 (352–665)	466 (318–624)	501.5 (347–672)	497 (371–706)	463.5 (406–718)	398 (257–568)	441 (302–613)	476.5 (349–652)	521.5 (368–689)

N: total study population; n: subset of study population; IQR: interquartile range; < : less than; ≥ : greater than or equals to.

### Overall viral load and CD4-cell count trends in those with and without hypertension

**Figure 2:**
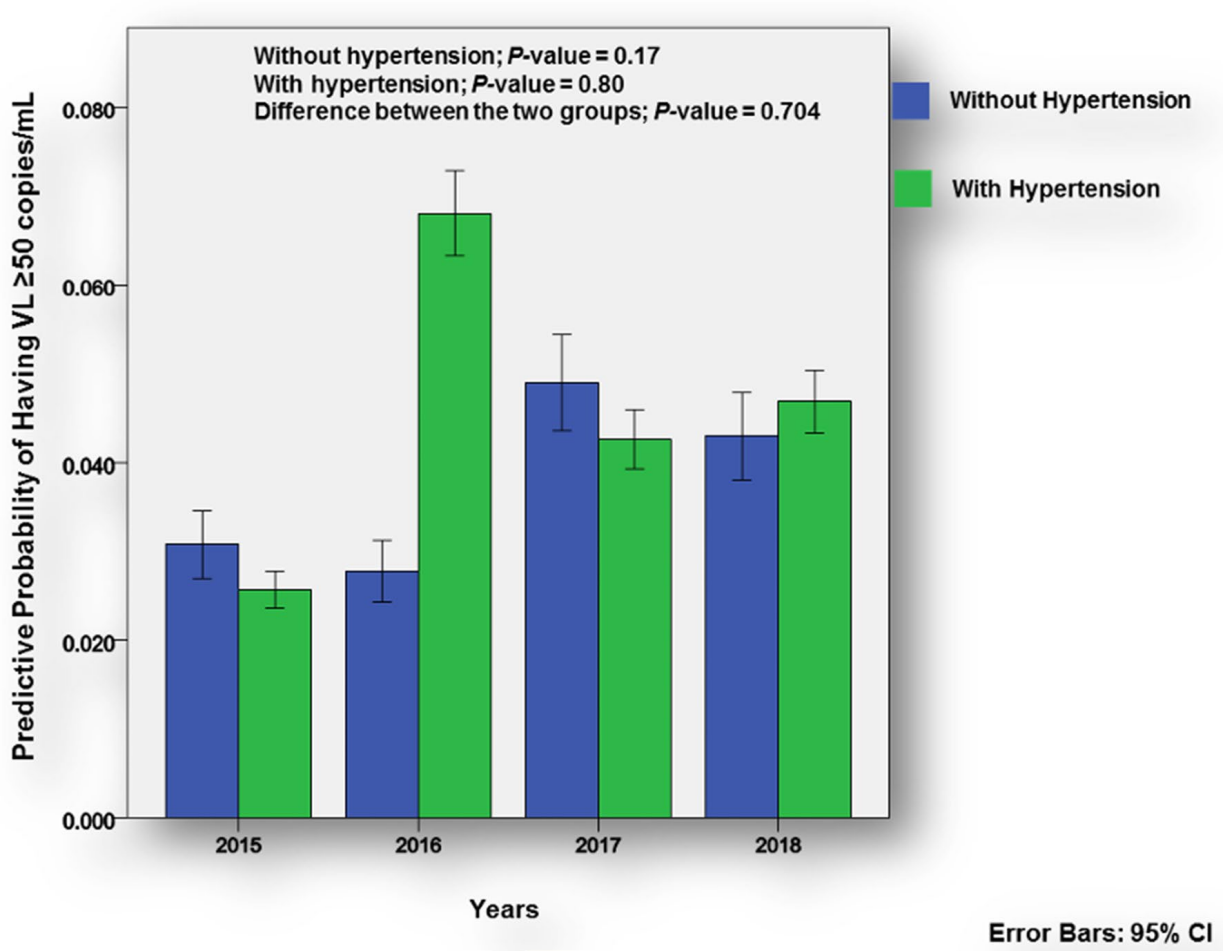
Predictive probability in relation to viral loads.

**Figure 3:**
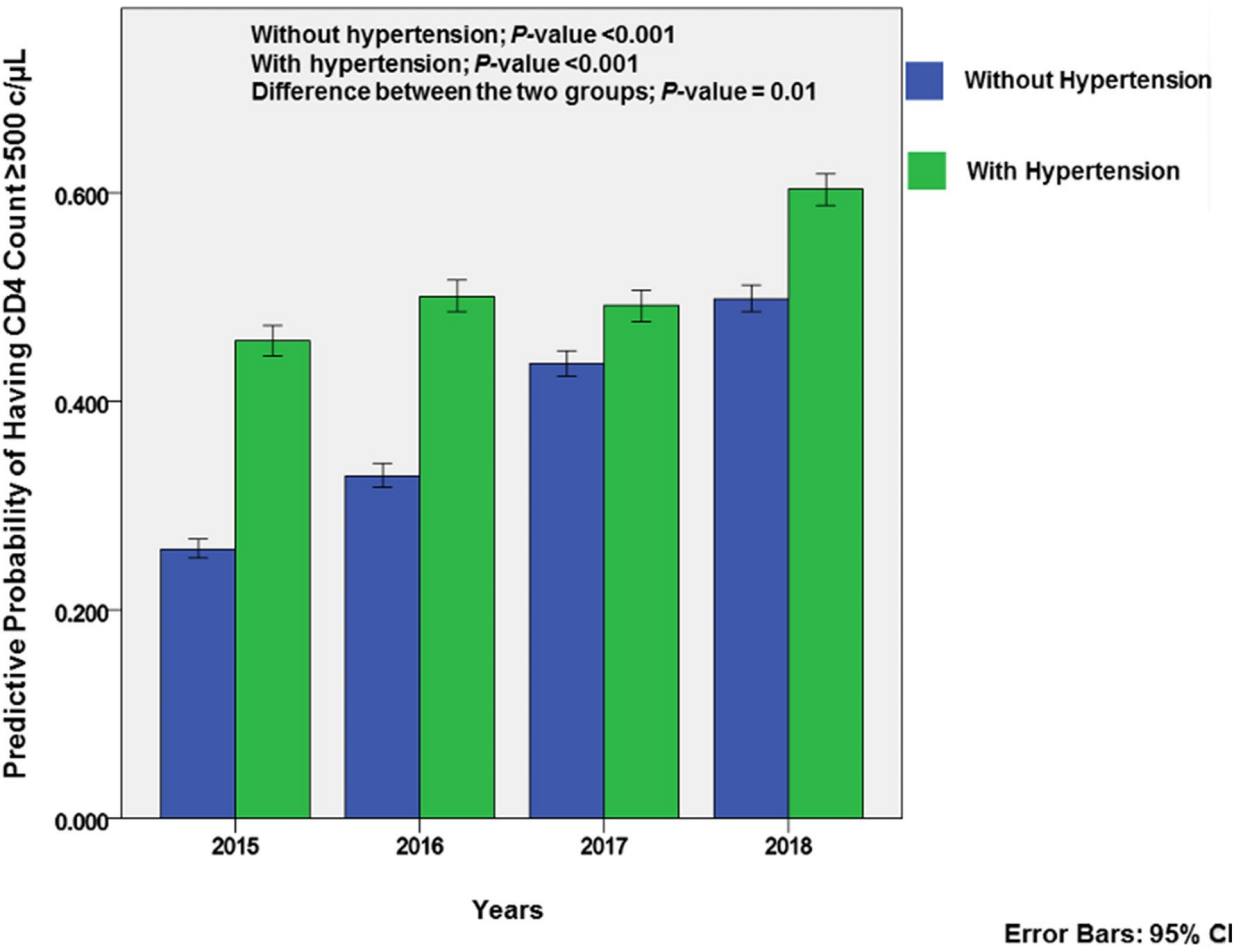
Predictive probability in relation to CD4-Cell counts.

According to Fig. 2, the overall trend from 2015 to 2018 that viral loads would be ≥ 50 copies/mL was not significant (*p-*value = 0.17). Hypertensive participants had a 2.6% (95% CI 2.4–2.8%) and 6.8% (95% CI 6.3–7.3%) probability of having viral loads of ≥ 50 copies/mL in 2015 and 2016, respectively. This increase was marginal and the overall trend was insignificant (*p-*value = 0.80). The difference between the two groups was also insignificant (*p-*value = 0.70). As can be seen in Fig. 3, there was significant change in probability of having CD4-cell counts ≥ 500 cells/µL in both groups over the study period (*p-*value < 0.001). There was also a significant difference in the increased probability between the two groups (*p-*value = 0.01) overall, except in 2017 when hypertensive participants showed only a slight decrease.

### Association between confounding factors and outcomes in all participants

A Generalized Estimated Equations model was used to study the association between possible confounding factors (hypertension, age, sex, residency, IPT, WHO HIV stage, ART line of treatment, adverse/side effects, drug substitution, adherence, and time) and our outcome variables. As can be seen in the whole-group analysis in Table 4, hypertensive participants were more likely to have detectable viral loads (OR = 1.37), after adjustment of other variables; although this was insignificant (95% CI 0.77–2.43). Participants receiving second line ART treatment were more likely to have a detectable viral load (OR = 7.91; 95% CI 2.53–24.68). The whole-group analysis in Table 5 shows that hypertensive participants were also more likely to have CD4-cell counts ≥ 500 cells/µL (OR = 1.83; 95% CI 1.37–2.44). There were other variables, including age, gender, IPT, viral load, and adherence significantly associated with CD4-cell counts. Patients 41–50 years old (vs. 30–40 years), males, and those with detectable viral loads were less likely to have CD4-cell counts of ≥ 500 cells/µL compared to their counterparts (OR = 0.73; 95% CI 0.532–0.997: OR = 0.48; 95% CI 0.36–0.64 and OR = 0.62; 95% CI 0.40–0.98, respectively). Participants with good adherence were more likely to have CD4-cell counts of ≥ 500 cells/µL (OR = 1.64; 95% CI 1.15–2.33). The year of study was also significantly related to the outcome variable, 2015 vs. 2016 (OR = 1.32; 95% CI 1.09–1.60), 2017 (OR = 1.71; 95% CI 1.40–2.09), and 2018 (OR = 2.44; 95% CI 2.00–2.98).

**Table 4:** Factors associated with viral load.

Parameters	All patients		Non-hypertensive		Hypertensive	
	OR (95% CI)	*p*-value	OR (95% CI)	*p*-value	OR (95% CI)	*p*-value
**Hypertension**						
No	1.00					
Yes	1.37 (0.77–2.43)	0.29				
**Age**						
30–40 years	1.00		1.00		1.00	
41–50	0.87 (0.49–1.55)	0.64	0.95 (0.43–2.10)	0.90	0.70 (0.27–1.85)	0.47
> 50	0.76 (0.36–1.60)	0.50	0.48 (0.13–1.79)	0.28	0.66 (0.22–1.97)	0.45
**Sex**						
Female	1.00		1.00		1.00	
Male	1.06 (0.61–1.83)	0.84	0.78 (0.37–1.67)	0.53	1.44 (0.62–3.33)	0.39
**IPT**						
No	1.00		1.00		1.00	
Yes	1.50 (0.85–2.64)	0.16	1.80 (0.84–3.86)	0.13	1.05 (0.44–2.51)	0.91
**WHO HIV stage**						
Stage 1	1.00		1.00		1.00	
Stage 2–4	1.31 (0.49–3.50)	0.59	0.34 (0.07–1.70)	0.19	2.84 (1.03–7.85)	0.04*
**ART adverse effects**						
No	1.00		1.00		1.00	
Yes	1.89 (0.73–4.86)	0.19	3.50 (1.06–11.54)	0.04﻿*	1.35 (0.38–4.73)	0.66
**ART line of treatment**						
1st line	1.00		1.00		1.00	
2nd line	7.91 (2.53–24.68)	﻿< 0.001*	8.61 (2.93–25.34)	<0.001 *		
**ART substitution**						
No	1.00		1.00		1.00	
Yes	1.73 (0.85–3.52)		2.22 (0.83–5.94)	0.11	1.33 (0.46–3.79)	0.60
**ART adherence**						
Poor adherence	1.00		1.00		1.00	
Excessive adherence	1.89 (0.78–4.61)	0.16	0.72 (0.21–2.46)	0.60	8.08 (1.33–49.04)	0.02*
Good adherence	0.80 (0.39–1.66)	0.55	0.45 (0.18–1.10)	0.08	2.75 (0.45–16.83)	0.26
**Study time**						
2015	1.00		1.00		1.00	
2016	1.61 (0.91–2.86)	0.10	0.89 (0.37–2.14)	0.80	2.85 (1.22–6.69)	0.02*
2017	1.68 (0.91–2.86)	1.10	1.69 (0.79–3.63)	0.18	1.71 (0.59–4.97)	0.32
2018	1.60 (0.84–3.07)	0.15	1.45 (0.62–3.42)	0.39	1.90 (0.66–5.43)	0.22

IPT: isoniazid preventive therapy; HIV: human immunodeficiency virus; > : greater than; CI: confidence interval; < : less than; OR: odds ratio; –: independent variable has collinearity with independent variable; 1.00: reference.

*Statistically significant P-value.

**Table 5:** Factors associated with CD-4 cell counts.

Parameters	All patients		Non-hypertensive		Hypertensive	
	OR (95% CI)	*p*-value	OR (95% CI)	*p*-value	OR (95% CI)	*p*-value
**Hypertension**						
No	1.00					
Yes	1.83 (1.37–2.44)	< 0.001*				
**Age (years)**						
30–40	1.00		1.00		1.00	
41–50	0.73 (0.532–0.997)	0.05*	0.88 (0.58–1.32)	0.53	0.61 (0.37–0.98)	0.05*
> 50	0.95 (0.66–1.38)	0.81	0.98 (0.54–1.77)	0.94	0.89 (0.54–1.45)	0.63
**Sex**						
Female	1.00		1.00		1.00	
Male	0.48 (0.36–0.64)	<0.001*	0.53 (0.36–0.78)	0.001*	0.36 (0.22–0.57)	<0.007 *
**Taken IPT**						
No	1.00		1.00		1.00	
Yes	1.53 (1.16–2.01)	0.001*	1.68 (1.16–2.45)	0.01*	1.44 (0.95–2.17)	0.09
**WHO HIV stage**						
Stage 1	1.00		1.00		1.00	
Stage 2–4	0.83 (0.44–1.53)	0.54	1.24 (0.40–3.81)	0.71	0.54 (0.24–1.20)	0.13
**ART line of treatment**						
1st line	1.00		1.00		1.00	
2nd line	0.73 (0.29–1.89)	0.54	0.54 (0.18–1.63)	0.28	4.67 (2.01–10.86)	<0.001 *
**ART adverse effects**						
No	1.00		1.00		1.00	
Yes	0.54 (0.26–1.11)	0.09	0.60 (0.16–2.27)	0.45	0.54 (0.22–1.37)	0.19
**Had ART substitution**						
No	1.00		1.00		1.00	
Yes	1.01 (0.64–1.59)	0.96	1.69 (0.87–3.29)	0.12	0.62 (0.35–1.09)	0.10
**ART adherence**						
Poor adherence	1.00		1.00		1.00	
Excessive adherence	1.47 (0.88–2.47)	0.14	0.77 (0.38–1.56)	0.47	4.03 (1.78–9.12)	0.001*
Good adherence	1.64 (1.15–2.33)	0.01*	1.37 (0.87–2.14)	0.17	2.15 (1.15–4.05)	0.02*
**Study time**						
2015	1.00		1.00		1.00	
2016	1.32 (1.09–1.60)	0.001*	1.43 (1.10–1.86)	0.01*	1.22 (0.90–1.67)	0.20
2017	1.71 (1.40–2.09)	<0.001*	2.37 (1.80–3.12)	<0.001 *	1.17 (0.85–1.60)	0.34
2018	2.44 (2.00–2.98)	<0.001*	3.09 (2.37–4.04	<0.001 *	1.92 (1.39–2.67)	<0.001 *
**Viral load**						
< 50 c/mL	1.00		1.00		1.00	
≥ 50 c/mL	0.62 (0.40–0.98)	0.04*	0.46 (0.25–0.84)	0.01*	0.77 (0.38–1.57)	0.48

IPT: isoniazid preventive therapy; HIV: human immunodeficiency virus; > : greater than; ≥ : greater than or equals to; CI: confidence interval; < : less than; OR: odds ratio; –: independent variable has colinearity with independent variable; 1.00: reference.

*Statistically significant P-value.

### Association between confounding factors and outcomes in non-hypertensive and hypertensive participants

As can be seen in the subgroup analysis in Table 4, in non-hypertensive participants, there was a significant association between viral load and adverse/side effects as well as line of ART (OR = 3.50; 95% CI 1.06–11.54 and OR = 8.61; 95% CI 2.93–25.34, respectively). In hypertensive participants, viral load was significantly associated with 2015 vs. 2016 time-point, HIV stage, and adherence (OR = 2.85; 95% CI 1.22–6.69: OR = 2.84; 95% CI 1.03–7.85 and OR = 8.08; 95% CI 1.33–49.04). The subgroup analysis in Table 5 shows a significant association between sex, IPT, time of study, as well as viral load and CD4-cell counts in non-hypertensive participants. In hypertensive participants, CD4-cell counts were significantly associated with age, gender, line of treatment, adherence, and time to some extent. In both subgroups, males were less likely to have CD4-cell counts ≥ 500 cells/µL (non-hypertensive, OR = 0.53; 95% CI 0.36–0.78; hypertensive, OR = 0.36; 95% CI 0.22–0.57).

## Discussion

In our analysis of all ART patients, the present study found a significant association between hypertension and CD4 cell counts but not viral load. This means that hypertensive patients were more likely to have improved CD4-cell counts compared to non-hypertensive patients. In non-hypertensive patients there was a significant association between improved CD4-cell counts and being female, IPT, undetectable viral load, and time of study. In hypertensive patients, the variables significantly associated with improved CD4-cell count were younger age, 2nd line of ART, better adherence, and time of study.

This study found an increase in CD4-cell counts over time in both groups of patients, as has been previously reported^40–42^. The hypertensive patients were found to be more likely to have higher CD4-cell counts compared to non-hypertensive patients. It is important to note that both patients had the same follow-up in the study and in hospital in general, not unless they feel the need to come to the hospital. Other studies found no significant difference in CD4-cell counts in ART patients with and without hypertension^43,44^. Our results could be explained by the possibility that patients being treated and counseled for hypertension may have better lifestyle habits as they attempt to bring it under control and this may consequently lead to better immune system functioning. For instance, patients with hypertension are expected not to engage in smoking, alcohol use or any other substance abuse, have restricted diet that is with less fats and sodium. However, it is interesting to note that people with hypertension usually have higher BMIs^45^. Koethe et al*.* have reported an association between pre-ART BMI and 12-month change in CD4-cell counts (*P-*value < 0.001) and concluded that a BMI indicative of threshold obesity predicted greater CD4-cell count gains at the beginning of ART^46^.

We also found male ART patients to be less likely to have CD4-cell count improvement than females in both groups. One study of 7,354 patients initiating ART between April 2004 and April 2010 in South Africa also found men on ART have less CD4-cell improvement than women^47^. However, a review of eight cohort studies of European populations reported that more women than men seroconverted to HIV, developed AIDS and died with higher CD4-cell counts^48^.

We also found that patients receiving IPT were more likely to have higher CD4-cell counts. IPT contributes significantly to prevent incidences of active TB among PLHIV^49,50^. Therefore, the combination of IPT and ART probably lead to a boost in immunity. We found this to be significant among those without hypertension but insignificant among those with hypertension, possibly suggesting an interaction between hypertension or hypertension medications and IPT.

We found patients with close adherence to prescriptions to be more likely to have higher CD4-cell counts than those under medication, similar to a longitudinal study in the Hunan and Hubei provinces of China^51^^.^ One prospective 12-month cohort study associated adherence levels of 100%, 80–90%, and 0–79% with CD4-cell count increases of 179, 159, and 53 cells/µL, respectively, (*p-*value < 0.001)^52^. In our subgroup analysis, overmedication was associated significantly with better CD4-cell counts, more particularly with hypertensive patients. Close adherence was also associated significantly with better CD4-cell counts, more particularly with hypertensive patients. However, based on the literature, close adherence is important for everyone, regardless of whether they have hypertension or not. Despite its seemingly lack of effect on immunity, overuse could possibly lead to increased drug toxicity in some patients.

We found patients receiving second line ART to have higher CD4-cell counts than those receiving first line treatment, especially among those with hypertension. Antihypertensive drugs could possibly affect effectiveness of ART. For example, two kinds of ART drugs, NNRTIs (like Nevirapine and Efavirenz) and PIs, are metabolized primarily by CYP34A in the CYP450 system, the same pathway is involved in the metabolism of the hypertension drugs indapamide, calcium channel blockers, and losartan^53^.

Patients with detectable viral loads were found to be less likely to have higher CD4-cell counts (OR = 0.62; 95% CI 0.40–0.98). This association was found to be more significant in those without hypertension (OR = 0.46; 95% CI 0.25–0.84), suggesting that the additional healthcare benefitted those patients with hypertension by improving their immune systems.

While age was not found to be associated with CD4-cell counts in the whole group analysis, we did find an association between age and increased and decreased CD4-cell counts in patients with hypertension in the subgroup analyis. Among these patients, those 41–50 years old were less likely to have higher CD4-cell counts than those being 30–40 years old. Healthy individuals also gradually become immunodeficient over the long term^54,55^. However, one observational study of HIV patients in Australia found no long-term decline in CD4-cell counts in ART patients^56^, suggesting that the level of immune recovery achieved during the first 5 years of treatment was sustained through long-term ART. It is possible that the presence of hypertension might contribute to decreasing CD4-cell counts in those who are older, though this would require further studies.

As there was no significant association of hypertension with viral load, this means hypertensive patients and non-hypertensive patients have about the same likelihood of achieving viral load suppression when they are on ART, after controlling for other confounding factors. In a cross-sectional study aimed to estimate the prevalence of hypertension and describing the characteristics of patients with hypertension infected by HIV/AIDS in the state of Pernambuco in Brazil, the results reveal that there was no difference between hypertensive and pre-hypertensive patients with regard to parameters related to HIV infection and treatment such as viral load. One then can conclude that having hypertension does not prove to be a predisposing factor for one to not achieve viral load suppression^43^.

One factor was found to have a significant association with viral load in the whole group analysis, and that was ART line of treatment. Patients on the 2nd line of treatment were more likely to have a detectable viral load, compared to patients on 1st line. In a systematic review and meta-analysis aimed to summarize reported rules and reasons for virological failure among people on 2nd line therapy in resource-limited setting, the researchers found that the cumulative pooled proportion of adult patients failing virologically was 21.8% at 6 months, 23.1% at 12 months, 26.7% at 24 months and 38% at 36 months^57^. For patients on 2nd line treatment to have more likelihood for detectable viral load is likely to be more related to poor adherence. As the 2nd line treatment is associated with more adverse effects compared to the 1st line^58^, that can possibly make patients on the 2nd line not to adhere appropriately on the treatment as a way to avoid the adverse effects, yet by doing so they increase the viral load. This also explain why in our subgroup analysis patients with adverse effects were more likely to have detectable viral load compared to those who experienced no or less adverse effects. This happened more particularly with the group of patients without hypertension (OR = 3.5; 95% CI 1.06–11.54).

Based on the changes or trend of viral load with time, the results in this study reveal fluctuating predictive probability of viral load to be detectable for hypertensive and non-hypertensive patients. For patients with hypertension, the predictive probability was more significantly higher in 2016 compared to the other years. The fact that the viral load temporary increased significantly in 2016 can be due to what is known as “blip” and is normal. It can also be possibly due to that the patients might have flue/cold or other short illness or they have just been vaccinated^59^. A “blip” is when the viral load increase above 50 copies/mL temporary and then drops back^60^.

The findings of the study can assist the country (Eswatini) and the southern African region at large by reflecting on the effectiveness of HIV/AIDS and non-communicable disease programs that are currently taking place in the country, in order to determine the country’s progress in achieving Sustainable Development Goal 3 of Good Health and Wellbeing, and the 90-90-90 world target set by UNAIDS in 2014. The findings can also illustrate the possible effectiveness of integrating HIV and non-communicable services. The research findings and recommendations also have a potential for contributing to and extending the scientific body of public health knowledge. They have a potential to facilitate amendments of existing guidelines dealing with HIV/AIDS care and treatment, and NCDs.

The study encountered some limitations. Firstly, it is based on a retrospective analysis of already collected data from chronic patient files. The variation of clients’ ART initiation and hypertension diagnosis and treatment initiation dates were factors that proved to be confounding for this study, hence a causal relationship could not be established, even though the study was longitudinal. Also, these variations made it hard for the researchers to collect important information related to variables such as BMI, as some files would not have all the necessary information needed to formulate such a variable as it would be missing. Therefore, the missing information pertaining to variables (such as BMI, socio-economic status, or alcohol use) proved to be the largest limitation for this study. However, efforts have been made to account for them in the discussion section, based on findings from other researchers.

## Conclusions

ART patients with hypertension were more likely to have higher CD4-cell counts compared to ART patients without hypertension in the whole group analysis. Underuse and overuse of medications, advanced HIV stages, adverse effects, line of treatment, age and sex predicted higher viral loads (ART failure) and lower CD4-cell counts in patients with hypertension. From such findings, one can conclude that patients with hypertension or with NCDs for that matter can also progress very well in treating HIV, more so if they observe their lifestyle habit and care for their NCDs treatment. More attention may need to be paid to ART patients with well controlled HIV status to monitor and control their hypertension status. Medical professionals, who may be overly concerned to control HIV/AIDs, may want to return their focus to the possible development of other chronic diseases, particularly hypertension, because these non-communicable diseases could later result in ART failure.

## Abbreviations

ALT: Alanine aminotransferase test
ART: Antiretroviral therapy
ARVs: Antiretrovirals
AST: Aspartate aminotransferase test
CKD: Chronic kidney disease
CYPs: Cytochromes P450
DBP: Diastolic blood pressure
EsMoH: Eswatini Ministry of Health
HAART: Highly active antiretroviral therapy
INH: Isonicotinylhydrazide (isoniazid)
IPT: Isoniazid preventive therapy
NCDs: Non-communicable diseases
NHRRB: National Health Research Review Board
NICE: National Institute for Health and Care Excellence
NNRTIs: Non-nucleoside reverse transcriptase inhibitors
NRTIs: Nucleoside reverse transcriptase inhibitors
PAGAA: Panel on antiretroviral guidelines for adults and adolescents
PAHO: Pan American Health Organization
PEPFAR: President’s emergency plan for AIDS relief
PIs: Protease inhibitors
PLHIV: People living with HIV
RFMH: Raleigh Fitkin Memorial Hospital
SAHCS: Southern African HIV Clinicians Society
SBP: Systolic blood pressure
SCr: Serum creatinine
SHIMS: Swaziland HIV Incidence Measurement Survey
SNAP: Eswatini National Program
